# Trend Analysis of Suicide and Homicide Mortality and Years of Life Lost (YLL) in Children Aged 10-19 Years in the South of Iran, 2004-2019

**DOI:** 10.34172/jrhs.2024.141

**Published:** 2024-03-10

**Authors:** Habibollah Azarbakhsh, Fatemeh Jafari, Seyed Parsa Dehghani, Andishe Hamedi, Mohammad Hossein Sharifi, Alireza Mirahmadizadeh

**Affiliations:** ^1^Department of Epidemiology, School of Health, Ahvaz Jundishapur University of Medical Sciences, Ahvaz, Iran; ^2^Student Research Committee, Shiraz University of Medical Sciences, Shiraz, Iran; ^3^Department of Social Medicine, School of Medicine, Shiraz University of Medical Sciences, Shiraz, Iran; ^4^Research Center for Traditional Medicine and History of Medicine, Shiraz University of Medical Sciences, Shiraz, Iran; ^5^Non-Communicable Diseases Research Center, Shiraz University of Medical Sciences, Shiraz, Iran

**Keywords:** Children, Suicide, Homicide, Years of life lost, Joinpoint regression, Iran

## Abstract

**Background:** This study aimed to investigate mortality and years of life lost (YLL) due to suicide and homicide in children aged 10-19 years in southern Iran from 2004 to 2019.

**Study Design:** A cross-sectional study.

**Methods:** The data on all deaths due to suicide and homicide in Fars province were obtained from the population-based electronic death registration system (EDRS). Crude mortality rate and YLL were calculated. The joinpoint regression method was used to examine the trend.

**Results:** During the study period, 563 cases of suicide and 218 cases of homicide in children aged 10-19 have occurred. The total number of YLL due to suicide was 9766 in men and 6261 in women. According to the joinpoint regression analysis, the trend of YLL due to suicide was increasing in males. In other words, the annual percent change (APC) was 4.8% (95% CI 0.4 to 9.5, *P*=0.036). Additionally, there was a constant trend in females, and APC was 2.7% (95% CI -2.0 to 7.7, *P*=0.241). The number of YLL due to homicide was 4890 in males and 1294 in females. The trend of YLL due to homicide was stable in males and females. In other words, APC was -1.6% (95% CI -5.6 to -2.6, *P*=0.422) in males and -2.7% (95% CI -10.0 to 5.2, *P*=0.467) in females.

**Conclusion:** Based on the findings of this study, the trend of mortality rate and YLL due to suicide in men has been increasing and it has been stable in women. Moreover, the trend of mortality due to homicide was stable for both males and females. Therefore, it is necessary to take preventive actions.

## Background

 Unnatural deaths, including deaths resulting from intentional injuries such as homicide or suicide, are among the major global public health concerns.^1^ This category of deaths is one of the leading causes of premature death, imposing a significant economic and societal burden on society.^2^ Approximately 800 000 suicides occur worldwide annually (one death every 40 seconds).^3,4^ The rate of suicide attempts is 10 to 20 times higher than completed suicides.^4^ Suicide attempts and suicidal behavior constitute the nineteenth leading cause of the global disease burden.^5^ The suicide rate varies across countries, ranging from 30 per 100 000 individuals per year in Russia to less than 1 per 100 000 individuals in Arab countries like Egypt.^6^ In Iran, a 20-year trend indicates a rise in suicide fatalities, with an average rate of 9.9 deaths per 100 000 individuals annually.^7^ Various studies have shown that the underlying factors contributing to suicide include personality traits (such as aggression), mental and physical disorders (like depression, pain, and disability), life events (such as the loss of friends or loved ones), social isolation, economic conditions, access to means of suicide, substance abuse, marital status, population density, birth rates, urban population, per capita income, unemployment rates, education, and religious affiliation.^6,8-10^

 In addition to suicide, intentional homicide has alarming statistics, with 470 000 people worldwide falling victim to homicide annually.^2,11^ Approximately half of these victims are 10 to 29 years old, and 84% of homicides occur among men.^2^ In Iran, the homicide rate is 6.5 per 100 000 individuals, with 80% of victims being male and an average age of 32.4 years.^2^ The World Health Organization (WHO) reports that suicide is one of the top three leading causes of death in the 15 to 44 age group,^12^ and homicide ranks as the fifth leading cause of death in the 10-14 age group.^11^ Therefore, adolescence and early adulthood bear the most significant burden of premature deaths due to suicide and homicide. In other words, reducing mortality rates in this population segment prepares society for a healthier and better future. Suicide and homicide rates vary from one country to another and across different age groups of a country.^6^ In a study conducted in Ilam province from 2014 to 2018, the homicide and suicide rates were reported to be 8.1 and 20.7 per 100 000 individuals, respectively, with the highest number of suicide attempts observed in individuals aged 15 to 24 years. Furthermore, the suicide and homicide rates in this age group (15-24 years) were 33.9 and 9.1 per 100 000 individuals, respectively.^2^ In a study conducted in Fars province during 2011-2018, the suicide rate was reported to be 9.68 per 100 000 individuals, with the highest number of years of life lost (YLL) observed in individuals aged 15 to 29.^12^ Based on the results of the study, the number of YLL due to homicide was reported to be 1.37 per 1000 individuals in males and 0.29 per 1000 individuals in females. Additionally, the highest number of YLL due to homicide in both genders was observed in the 15-29 age group.^11^ Various factors, including socioeconomic pressures, cultural factors, and literacy, play a role in the occurrence of homicides.^11^

 Examining these phenomena in different regions is important because different communities may be exposed to these events with varying conditions and risk factors. Additionally, calculating YLL serves as a fundamental measure for ranking the overall health status of a society and identifying its challenges to raise awareness and implement preventive measures.^11^ YLL, defined as the number of years of death from a disease prior to death in the absence of that disease, is a measure of disease burden on the general population.^13^ Therefore, this study aims to provide details on mortality and YLL due to suicide and homicide in individuals aged 10-19 years in southern Iran from 2004 to 2019.

## Materials and Methods

 This cross-sectional study was conducted in Fars province from 2004 to 2019. The data on all suicide and homicide deaths were extracted from the population-based electronic death registration system (EDRS) in the age group of 10-19 years and according to age, gender, and year of death based on ICD-10. The ICD-10 codes used in this study were X60-X84 for suicide and X85-Y09 for homicide.

 The entry criteria included being 10-19 years of age and being a resident of Fars province. The total population of Fars province has been estimated using the basic data of health centers and the population and housing census from 1996 to 2016, taking into account the annual growth of the population. For standardization, the standard population of 2013 for countries with low and moderate incomes was used. Compared to this standard population, the standard population of the WHO has a lower proportion of the younger population and a higher proportion of those over 70 years old. As a result, it is not suitable for low- and middle-income countries where the proportion of young people is higher.^14^

## Statistical analysis

 First, descriptive statistics were reported as numbers and percentages. Afterwards, the crude mortality rates of suicide and homicide were calculated during the years of the study according to gender and year of death.

 Then, YLL was calculated using the standard life table and determining life expectancy for different age (10-19 years) and gender groups, as well as the number of deaths due to suicide and homicide, in each age and gender group, based on the following equation.^15^


$$YLL=N\;Ce^{\left(ra\right)}/{\left(\beta +r\right)}^{2}\left[e^{-\left(\beta +r\right)\;\left(L+a\right)}\left[-\left(\beta +r\right)\left(L+a\right)-1\right]-e^{-\left(\beta +r\right)\;a}\left[-\left(\beta +r\right)a-1\right]\right]$$

Which *N* is the number of deaths in a certain age and gender group; *L* is the standard life expectancy of the deceased people in the same age group and gender (males aged 10-14, females aged 10-14, males aged 15-19, and females aged 15-19); *r* is the discounting rate, which is equal to 0.03; *β* is the contractual rate in calculating the age value, which is equal to 0.04; *C* = an adjusted fixed value, which is equal to 0.1658; *a* = age at which death occurred; and *e* is constant and equal to 2.71

 The analysis of the number of years of life lost due to suicide and homicide was done using the YLL template of 2015, WHO, in Excel spreadsheet version 2016.

 Then, to examine the trend of crude mortality rate and YLL, joinpoint regression was used. Joinpoint regression analysis describes the trend of change in successive segments of time and the decrease or increase within each segment.^3^ To determine the direction and magnitude of recent trends, annual percentage change (APC) and average annual percentage change (AAPC) and their 95% confidence intervals (CIs) for the last 16 available years were evaluated. AAPC was calculated as the weighted geometric mean of different APCs from joinpoint regression analysis, with weights equal to the length of each segment during the specified time interval.^16^ A joinpoint of zero indicates a straight line, and the optimal number of joinpoints was identified using the Monte Carlo permutation method.^17^ Unlike linear regression which is based on slope, the log linear regression is based on the APC (i.e., the rates change at a constant percent per year). It can also be used to compare trends across scales.^18^ To estimate the APC, the following equation was used^19^:


$$APC=\frac{e^{b0+b1\left(x+1\right)}-e^{b0+b1x}}{e^{b0+b1x}}\times 100=\left(e^{b1}-1\right)\times 100$$

When there are no joinpoints (i.e., no changes in trend), APC is constant, so it equals the AAPC. Otherwise, the whole period is segmented by the points with trend change.

 An approximate 95% confidence interval is used for AAPC in AAPCL and AAPCU formulas:


$$\begin{array}{l} AAPC_{L}=\left(e^{\log\left(AAPC+1\right)-1.96\sqrt{w_{x}^{2}\;\delta_{x}^{2}}}-1\right)\times 100\\ AAPC_{U}=\left(e^{\log\left(AAPC+1\right)+1.96\sqrt{w_{x}^{2}\;\delta_{x}^{2}}}-1\right)\times 100 \end{array}$$

Joinpoint Regression Program 4.9.1.0 was used to carry out the analysis of the trend.

## Results

 During the 16 years of the study (2004-2019), 563 cases of suicide and 218 cases of homicide in children aged 10-19 have occurred in Fars province. Based on the results, 61.3% of deaths caused by suicide and 79.4 % of deaths caused by homicide were observed in men.

 The highest number of deaths in both genders occurred in the age group of 15-19 years (Table 1).

**Table 1:** Number of deaths, the mortality rate (per 100­000 population), and years of life lost due to suicide and homicide in children according to age groups and gender in Fars province during 2004-2019

**Age group**	**Number of deaths**			**Mortality rate per 100­000**			**Years of life lost**		
	**Male**	**Female**	**Total**	**Male**	**Female**	**Total**	**Male**	**Female**	**Total**
Suicide									
10-14	49	34	83	2.0	1.5	1.7	1418	996	2414
15-19	296	184	480	10.1	6.6	8.4	8348	5265	13613
Total	345	218	563	6.4	4.2	5.4	9766	6261	16027
Homicide									
10-14	15	10	25	0.6	0.4	0.5	434	293	727
15-19	158	35	193	5.4	1.2	3.4	4456	1001	5457
Total	173	45	218	3.2	0.9	2.1	4890	1294	6184

**Figure 1:**
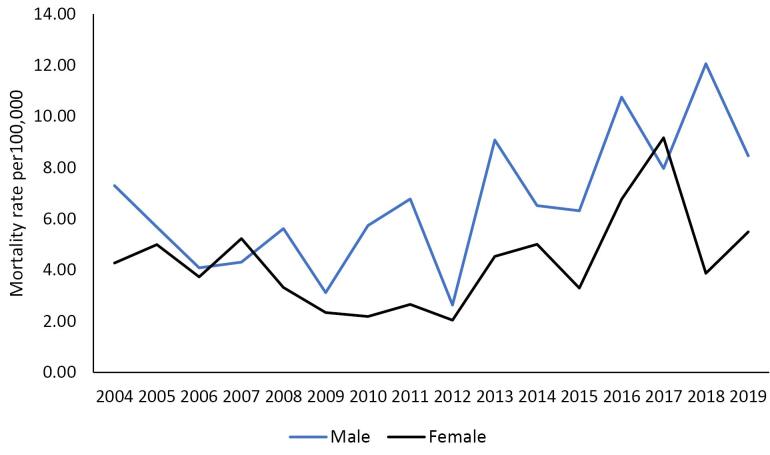
The mortality rate due to suicide in children in Fars province during 2004-2019

 As can be seen in Figure 1, the crude mortality rate of suicide in men increased from 7.3 (per 100 000 population) in 2004 to 8.5 (per 100 000 population) in 2019 (*P* for trend = 0.030, AAPC = 4.9), and in women, it increased from 3.4 (per 100 000 population) in 2004 to 5.5 (per 100 000 population) in 2019 (*P* for trend = 0.241, AAPC = 2.8) (Figure 1).

**Figure 2:**
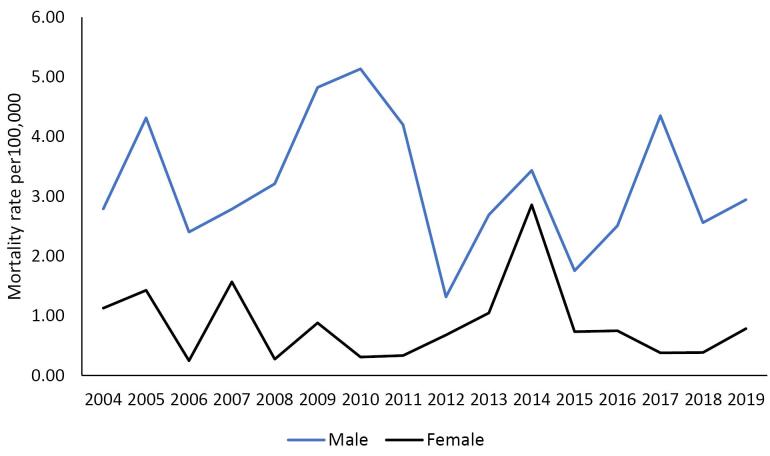
The mortality rate due to homicide in children in Fars province during 2004-2019

 Moreover, the crude mortality rate of homicide in men increased from 2.8 (per 100 000 population) in 2004 to 2.9 (per 100 000 population) in 2019 (*P* for trend = 0.500, AAPC = -1.4), and in women, it decreased from 1.1 (per 100 000 population) in 2004 to 0.8 (per 100 000 population) in 2019 (*P* for trend = 0.903, AAPC = -0.5) (Figure 2).

 The total number of years of life lost due to suicide during the 16-year study period was 9766 (1.8 per 1000 people) in men, 6261 (1.2 per 1000 people) in women, and 16027 (1.5 per 1000 people) in both genders (male/female ratio, 1.6) (Table 1). Besides, the total number of years of life lost due to homicide was 4890 (0.9 per 1000 people) in men, 1294 (0.3 per 1000 people) in women, and 6184 (0.6 per 1000 people) in both genders (male/female ratio, 3.8) (Table 1).

 The highest number of years of life lost due to suicide, according to the method of suicide, belonged to hanging (54.2%) and poisons and chemicals (10.4%) in men and self-immolation (36.2%) and hanging in women (23.9%) (Table 2).

**Table 2:** Years of Life lost due to suicide in children according to gender and method of suicide in Fars province during 2004-2019

**Method of suicide**	**Number of deaths**			**Years of life lost**			**YLL ratio**	**% total YLL**
	**Male**	**Female**	**Total**	**Male**	**Female**	**Total**	**M/F ratio**	
Hanging	187	52	239	5298	1497	6795	3.5	42.4
Drug overdose	27	29	56	761	836	1597	0.9	10.0
Toxic agent	36	32	68	1018	917	1935	1.1	12.1
Self-immolation	30	79	109	850	2264	3114	0.4	19.4
Firearms	28	9	37	791	258	1049	3.1	6.5
Drowning	1	2	3	28	57	85	0.5	0.5
Falling	3	2	5	85	58	143	1.5	0.9
Others	33	13	46	935	374	1309	2.5	8.2
Total	345	218	563	9766	6261	16027	1.6	100.0

**Figure 3:**
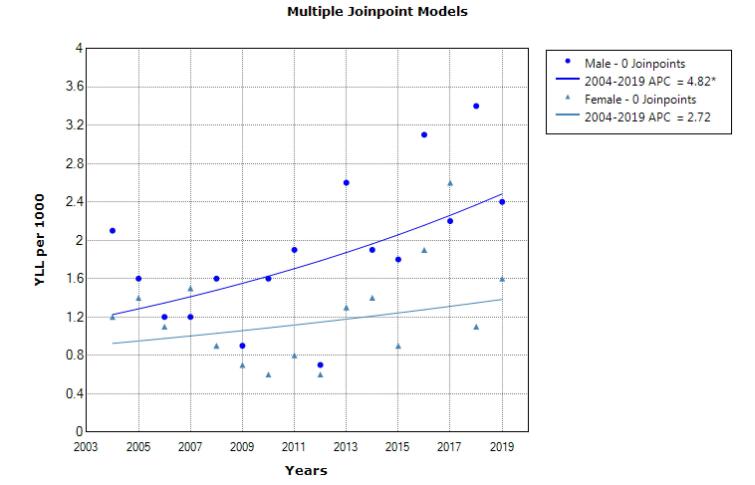
The trend of the number of years of life lost due to suicide in children aged 10-19 between 2004 and 2019

**Figure 4:**
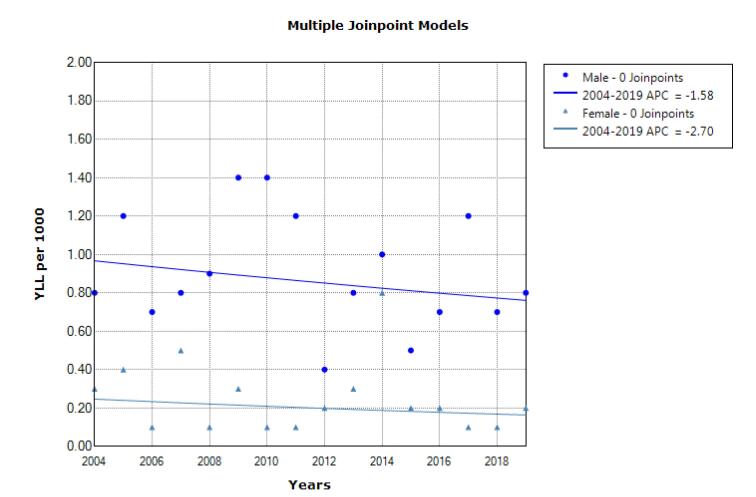
The trend of the number of years of life lost due to homicide in children aged 10-19 between 2004 and 2019

 According to the joinpoint regression analysis, the 16-year trend of YLL due to suicide was increasing in males; in other words, the annual percent change (APC) was 4.8% (95% CI 0.4 to 9.5, *P* = 0.036). In addition, stable trends were observed in females, and APC was 2.7% (95% CI -2.0 to 7.7, *P* = 0.241). The model did not show any join point; hence, the AAPC (Average Annual Percent Change) is the same as the APC (Figure 3). In addition, the trend of YLL due to homicide was stable, and APC was -1.6% (95% CI -5.6 to -2.6, *P* = 0.422) in males and it was -2.7% (95% CI -10.0 to 5.2, *P* = 0.467) in females. The model did not show any join point; hence, the AAPC is the same as the APC (Figure 4).

## Discussion

 This study was carried out to present the death rate due to suicide and homicide and to calculate the YLL of them. The results demonstrated that 563 cases of suicide and 218 cases of homicide occurred in children aged 10-19 in Fars province. School problems play a significant role in youth suicide, and many of the men and women who commit suicide are described by family, friends, and other acquaintances as sad and depressed. One study found that among suicide survivors with school problems (25% of the total), nearly one in eight had recently been bullied.^20^ In general, there is a complex interaction of mental health and school stressors, and several opportunities for intervention and prevention of suicidal behavior are suggested. For example, limiting physical access to devices is important in suicide prevention activities and it has been shown to be effective in a number of studies.^21,22^ Advances in various aspects of health care, including the development of emergency and trauma care systems, may play an important role in reducing homicide-related deaths.^23^

 In a study that examined the suicide status in 29 countries, it was shown that the rate of suicide in men is higher than in women, and it is higher in the age group of 15-19 years compared to the age group of 10-14 years,^24^ which is in line with the results of the present study. Suicide data found in China showed that suicide rates are higher among young women than young men, contrary to what is seen in Western societies.^25^ The higher rate of death due to suicide in the age group of 15-19 years justified that there are more mental problems, such as substance abuse.^26^ Maturity or university entrance exams can also play a significant role.^27^ It is also possible that other factors related to gender, such as aggression and drug use, play a role in increasing the suicide rate of men in some areas.^28^ In Brazil, the highest rate of mortality due to homicide was observed in the age group of 15-19 years and the rate of mortality was higher in men compared to women.^29^ Another reason for the higher number of deaths in men and people aged 15 to 29 is that they are in a more stressful situation than other groups. Additionally, they may have differences in attitude towards suicide.^2^

 It can be concluded that the trend of death due to suicide is increasing in both genders. The same result was obtained in Korea and Japan. On the other hand, in some European countries, a decrease in the death rate was observed in men and an increase in women. Economic stagnation, inequality, and rapid changes in family structure may be effective factors in this process.^24,30-32^ Several studies have offered explanations for this trend, such as restrictions on firearms and some degree of improvement in global health.^33,34^ Regarding the rate of homicide deaths, in the study conducted by Canudas-Romo and Aburto, a decreasing trend was observed.^35^ In our study, a small increase in death rate was observed in men and a decrease in women.

 A study conducted in Australia showed that in the age group of 15-19 years, the number of YLL was higher in women than in men,^36^ while in our study, for the entire period and both age groups, it was higher in men than in women for both suicide and homicide.

 In our study, the increase in the trend of YLL due to suicide was significant in men but it was not significant in women, and the increase in the trend of YLL due to homicide was not significant in either gender. This is an indication that there has been suicide in men at a younger age in recent years, which is a warning for families, schools, and health officials to pay more attention to this group and investigate the reasons. The reasons include unhealthy friendships, parental quarrels, parents’ lack of attention to the child or excessive attention, and cyberspace. Homicide has different trends in various regions depending on the prevailing conditions in that society. For example, in the study conducted by Ruch et al in the United States, the trend of death caused by suicide was first increasing, then decreasing, and then increasing.^37^ In the study conducted by Veisani et al in Ilam, the trend of death due to suicide was first decreasing and then increasing. The rate of mortality due to homicide was highest in 2017 and then it decreased.^2^ In the study conducted by Ruch et al, the chosen approach of young women to commit suicide had changed towards the more violent and deadly method of hanging and suffocation, while in our study, the most commonly used method was self-immolation in women and hanging in men.^37^ In another study, hanging and firearms were the top two methods used in suicide attempts in both genders.^20^ It should be noted that a possible reason for the higher number of YLL in men is that men tend to use more lethal methods.^28^ In some parts of our country, women may be more prone to self-immolation due to certain cultural reasons. Future studies are suggested to investigate the reasons for this.

 Our results revealed the prominent contribution of the male gender and individuals aged 15-29 years to the YLL. Considering the preventability of most of these types of deaths, there is a national need to implement an effective intervention in health policies to get rid of the burden of suicide in Fars province. Reducing youth suicide requires a multifaceted approach. However, a number of systematic literature reviews have been conducted indicating that effective interventions for self-injurious behaviors in youth are largely insufficient.^38-40^ Therefore, a new approach to suicide and homicide prevention is needed, with a strong national orientation supported by comprehensive and coordinated planning and implementation at the regional level.

 The strength of the study is that it calculated YLL and mortality from intentional injuries over a long period of time using joinpoint regression in order to identify changes made to this index. Death records are mandatory in all provinces of Iran and as a result, the missing data in the study was minimal. One of the limitations of this study is that there may be incorrect encoding when deaths are recorded, leading to incorrect classification. The study also looked at a specific age group, and if all age groups were considered, we might see a lot of changes in the results.

**Highlights**

- During the study period (2004-2019), 563 cases of suicide and 218 cases of homicide in children aged 10-19 have occurred.
- The total number of YLL due to suicide was 9766 in men and 6261 in women and the total number of YLL due to homicide was 4890 in men and 1294 in women.
- According to the joinpoint regression analysis, the 16-year trend of YLL due to suicide was increasing in men and stable in women.
- According to the joinpoint regression analysis, the 16-year trend of YLL due to homicide was stable in both genders.

## Conclusion

 Based on the findings of our study, the trend of mortality due to suicide is increasing in males and stable in females. Additionally, the number of YLL due to suicide has been increasing in men, and it has been stable in women. Moreover, the trend of mortality due to homicide was stable for both males and females. Therefore, it is necessary to take preventive actions, especially in men and vulnerable groups.

## Acknowledgments

 We would like to acknowledge the Health Vice-chancellor of Shiraz University of Medical Sciences.

## Authors’ Contribution

**Conceptualization:** Habibollah Azarbakhsh, Alireza Mirahmadizadeh.

**Data curation:** Fatemeh Jafari, Andishe Hamedi.

**Formal analysis:** Habibollah Azarbakhsh.

**Funding acquisition:** Alireza Mirahmadizadeh.

**Investigation:** Alireza Mirahmadizadeh

**Methodology:** Habibollah Azarbakhsh.

**Project administration:** Seyed Parsa Dehghani, Mohammad Hossein Sharifi.

**Software:** Habibollah Azarbakhsh.

**Supervision:** Habibollah Azarbakhsh.

**Validation:** Habibollah Azarbakhsh, Fatemeh Jafari, Andishe Hamedi.

**Visualization:** Habibollah Azarbakhsh, Mohammad Hossein Sharifi.

**Writing–original draft:** Habibollah Azarbakhsh, Fatemeh Jafari, Andishe Hamedi, Alireza Mirahmadizadeh.

**Writing–review & editing:** Habibollah Azarbakhsh, Fatemeh Jafari, Andishe Hamedi, Seyed Parsa Dehghani, Mohammad Hossein Sharifi Alireza Mirahmadizadeh.

## Competing Interests

 The authors declare that they have no conflict of interests.

## Ethical Approval

 The protocol of this study was reviewed and approved by the Ethics Committee of Shiraz University of Medical Sciences (IR.SUMS.REC.1399.772).

## Funding

 None.
